# Development of a Novel Enzyme-Targeting Radiosensitizer (New KORTUC) Using a Gelatin-Based Hydrogel Instead of a Sodium Hyaluronate

**DOI:** 10.3390/cancers8010010

**Published:** 2016-01-07

**Authors:** Shiho Morita-Tokuhiro, Yasuhiro Ogawa, Norikazu Yokota, Akira Tsuzuki, Hideki Oda, Naoya Ishida, Nobutaka Aoyama, Akihito Nishioka

**Affiliations:** 1 Department of Diagnostic Radiology & Radiation Oncology, Medical school, Kochi University, Nankoku, Kochi 783-8505, Japan; jm-tokuhiros@kochi-u.ac.jp (S.M.T.); jm-aoyama_nobutaka@kochi-u.ac.jp (N.A.); nishiokaa@kochi-u.ac.jp (A.N.); 2 Hyogo Prefectural Kakogawa Medical Center, Kakogawa, Hyogo 675-8555, Japan; 3 Division of Radiology, Kochi Medical School Hospital, Medical School, Kochi University, Nankoku, Kochi 783-8505, Japan; jm-yokotan@kochi-u.ac.jp (N.Y.); jm-tsuzukia@kochi-u.ac.jp (A.T.); 4 Medical School, Kochi University, Nankoku, Kochi 783-8505, Japan; jm-oda@kochi-u.ac.jp (H.O.); jm-ishidan@kochi-u.ac.jp (N.I.)

**Keywords:** radiosensitizer, hydrogen peroxide, KORTUC, sodium hyaluronate, hydrogel

## Abstract

We recently developed Kochi Oxydol-Radiation Therapy for Unresectable Carcinomas (KORTUC) as a strategy to increase intratumoral oxygen concentrations and degrade antioxidant enzymes such as peroxidase and catalase. We then developed KORTUC II, which uses sodium hyaluronate containing hydrogen peroxide as a radiosensitizer. KORTUC II requires twice-weekly administration to sustain its effects, but decreasing the frequency of radiosensitizer injections to once-weekly would reduce the burden on the patients and the physicians. The goal of this study was thus to develop a new formulation of KORTUC (New KORTUC) that only requires once-weekly administration. We performed experimental studies using a mouse tumor model and biodegradable hydrogel. C3H/He mice were allocated to control, KORTUC, or hydrogel groups. At 72 h after injection, each tumor was irradiated with a 6 MeV electron beam to a total dose of 30 Gy. During a 62-day observation period, changes in tumor volume and survival rates were assessed in each group. Tumor growth rate was slowest in the hydrogel groups. These data suggest that hydrogel could represent a useful adjunct as a long-acting radiosensitizer in place of sodium hyaluronate. New KORTUC, which contains hydrogen peroxide and hydrogel, exerted a radiosensitizing effect that persisted beyond 72 h following injection of the agent. Use of this new formulation allows radiosensitizer injections to be performed once-weekly with good effect.

## 1. Introduction

Low-linear energy transfer (LET) radiation, including X-rays and electron beams from linear accelerators, is used worldwide for clinical radiotherapy. However, the therapeutic effect of radiotherapy using linear accelerators are lower for large tumors (*i.e.*, exceeding several centimeters in diameter) than for small tumors. This is due to the presence of hypoxic tumor cells and an abundance of antioxidative enzymes, including peroxidase and catalase, in larger tumors [1]. Approximately two-thirds of the therapeutic effect attributable to linear accelerators is due to the indirect effects of X-rays and/or electrons, which generate hydroxyl radicals in the cytoplasm of tumor cells via radiation-induced degradation of cellular molecules and water [1]. The relative absence of oxygen in larger tumor tissues attenuates the anti-tumor effects of X-rays and electrons. Under these situations, use of a low concentration of hydrogen peroxide for low-LET-radioresistant neoplasms inactivates antioxidant enzymes such as peroxidase and catalase in tumor tissues, and generates oxygen via the degradation of hydrogen peroxide, thereby resulting in reoxygenation of the formerly hypoxic tumor.

We recently developed Kochi Oxydol-Radiation Therapy for Unresectable Carcinomas (KORTUC) as a method to increase intratumoral oxygen concentrations and inactivate antioxidative enzymes such as peroxidase and catalase [2,3,4,5,6]. KORTUC I, which uses hydrogen peroxide as a radiosensitizer, has demonstrated therapeutic efficacy against superficially exposed and locally advanced radioresistant neoplasms [7]. We next developed KORTUC II, which uses sodium hyaluronate containing hydrogen peroxide as a radiosensitizer for intratumoral injection [8,9]. The safety and efficacy of this therapy has been confirmed in clinical studies of patients with soft-tissue neoplasms, locally advanced breast cancer, early-stages breast cancer, and locally advanced pancreatic cancer [10,11,12,13,14,15,16]. KORTUC II inactivates peroxidase/catalase through the action of hydrogen peroxide and maintains intratumoral oxygen concentrations by the viscosity of sodium hyaluronate.

Tumor oxygen concentrations can be maintained for at least 24 h following the intratumoral injection of KORTUC II [8], and twice-weekly administration of this agent is necessary to achieve therapeutic effects. Twice-weekly injection represents a physical and mental burden to patients, and also a time and physical burden to doctors involved in KORTUC and radiological technologists conducting treatments. Development of an agent that can be administered once-weekly would reduce that burden. The goal of this study was therefore to develop a new formulation of KORTUC (New KORTUC) requiring only once-weekly administration. We performed experimental studies using a mouse tumor model and biodegradable hydrogel.

## 2. Results

**Figure 1:**
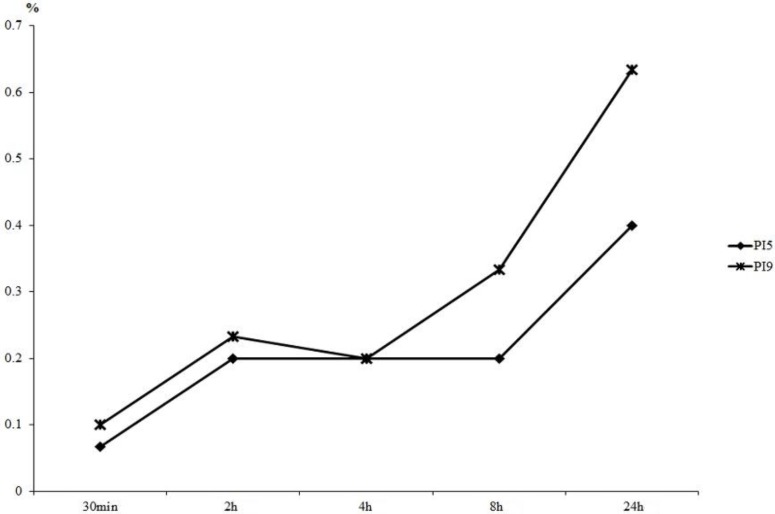
Measurement of hydrogen peroxide concentration released into PBS. By 4 h, little difference was seen between the hydrogen peroxide released from PI5 and PI9. Differences were seen at later time points, and the value of PI5 was two-thirds that of PI9 by 24 h.

Figure 1 shows the results of hydrogen peroxide released into buffer. Little difference in the concentration of hydrogen peroxide released was evident between PI5 and PI9 prior to 4 h. Differences subsequently began to appear gradually, and the value of PI5 eventually reached two-thirds that of PI9.

**Figure 2:**
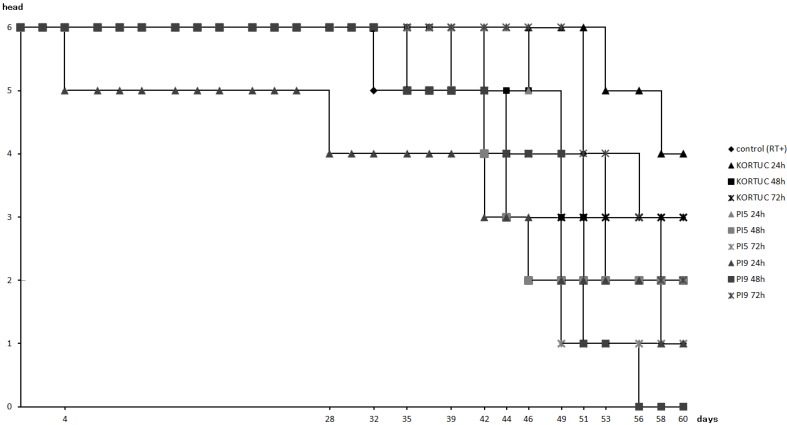
Survival rates of all groups. Survival rate was highest in the KORTUC (24 h) group (67%), followed by that in the KORTUC (72 h) group (50%). Survival rate was lowest (0%) in the PI9 (48 h) group. In addition, overall survival rate was 33% for four groups, and 16% for another three groups.

Figure 2 shows survival rates of all animal groups. Survival rate was highest in the KORTUC (24 h) group (67%). followed by the KORTUC (72 h) group (50%), while that of the PI9 (48 h) group was lowest (0%). In the other groups, survival rates of the KORTUC (48 h), PI5 (24 h), PI5 (48 h), and PI9 (72 h) groups were about 33%, and those of the control (RT+), PI5 (72 h), and PI9 (24 h) groups were about 16%.

**Figure 3:**
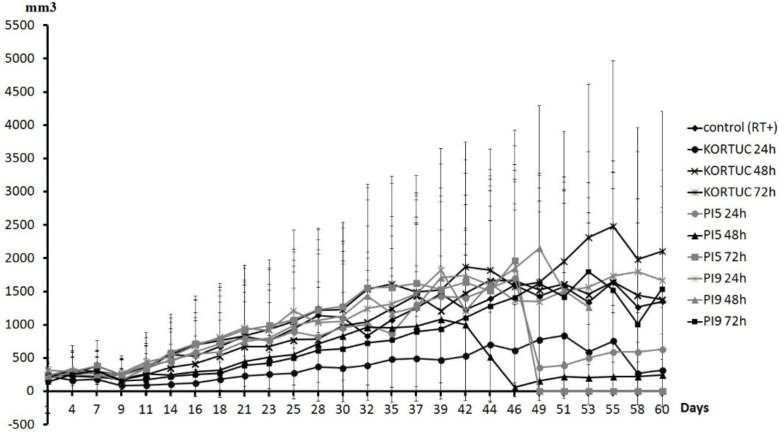
Tumor volume curve for all groups. Tumor growth rate was slowest in the KORTUC (24 h) group. The KORTUC (72 h) group did not show an inhibitory effect on tumor growth rate. During the observation period, complete resolution of tumors occurred in each group. Mice in which the tumors had disappeared on day 60 included: three mice in the KORTUC (24 h) group and one mouse each in the PI5 (72 h) and PI9 (72 h) groups.

Figure 3 shows tumor volume curves of all groups. Tumor growth rate was slowest in the KORTUC (24 h) group. The KORTUC (72 h) group did not show an inhibitory effect on the tumor growth rate. During the observation period, complete resolution of tumors occurred in some mice as follows. In the KORTUC (24 h) group, complete resolution of tumors occurred in four mice (on day 9 in one mouse, on day 11 in two mice, and on day 14 in one mouse), one of which showed tumor recurrence on day 21. In the KORTUC (48 h) group, resolution occurred in one mouse on day 21, with recurrence on day 30. In the PI5 (24 h) group, resolution occurred in two mice (on day 14 in one mouse, on day 16 in the other), one of which showed recurrence on day 32. In the PI5 (48 h) group, resolution occurred in two mice (on day 11 in one mouse, on day 14 in the other), one of which experienced tumor recurrence on day 39. In the PI5 (72 h) group, resolution occurred in one mouse on day 11. In the PI9 (24 h) group, resolution occurred in one mouse on day 9, with recurrence on day 28. In the PI9 (48 h) group, resolution occurred in one mouse on day 9, with recurrence on day 21. In the PI9 (72 h) group, resolution occurred in one mouse on day 21. Tumors had disappeared by day 60 in three mice in the KORTUC (24 h) group, and one mouse each in the PI5 (72 h) and PI9 (72 h) groups.

**Figure 4:**
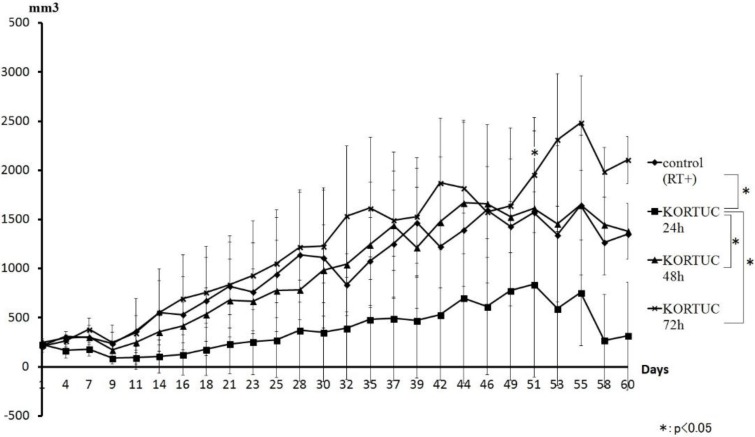
Comparison of the tumor volume curves of between the KORTUC and control groups. The KORTUC (24 h) group showed inhibition of increases in tumor volume compared with the other groups. For groups with a greater time to irradiation, no inhibition of tumor volume was identified. The KORTUC (72 h) group did not show inhibition of increases in tumor volume compared with the other groups. During the observation period, complete resolution of tumors occurred in the KORTUC group.

Figure 4 shows a comparison of the tumor volume curves of the KORTUC and control groups. The KORTUC (24 h) group showed inhibited tumor volume growth compared with the other groups. Moreover, increased inhibition of tumor volume did not occur with increases in the time to irradiation.

**Figure 5:**
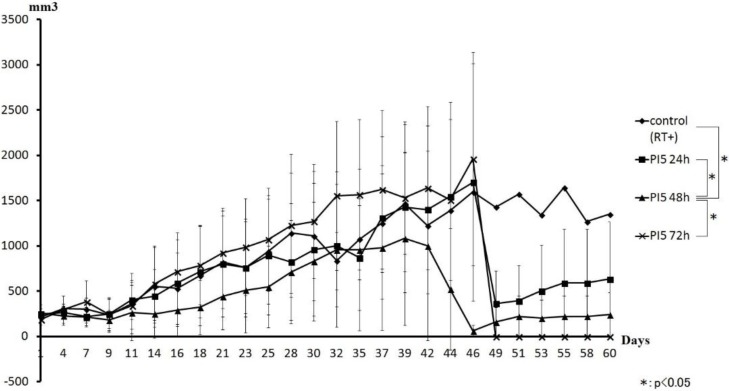
Comparison of the tumor volume curve between the PI5 and control groups. An inhibitory effect was seen in the group irradiated after 48 h. Abrupt decreases in tumor volume were identified on days 46 and 49. During the observation period, complete resolution of tumors occurred in the PI5 group.

Figure 5 shows a comparison of tumor volume curves for the PI5 and control groups. The PI5 (48 h) group showed inhibited tumor volume growth compared with the control groups. Abrupt decreases in tumor volume were seen at days 46 and 49.

**Figure 6:**
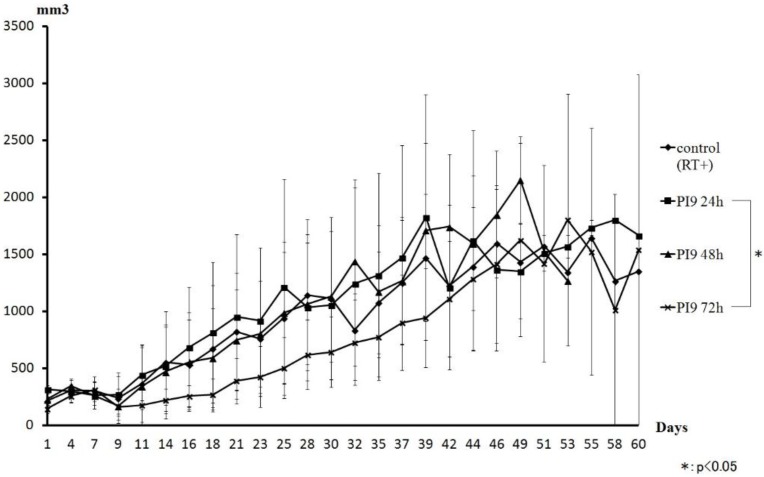
Comparison of the tumor volume curve for the PI9 and control groups. An inhibitory effect was seen in the group irradiated after 72 h. However, that group showed almost the same change as other groups on and after day 42. The PI9 (24 h) and PI9 (48 h) groups showed almost the same transition as the control group. During the observation period, the PI5 groups showed disappearance of mouse tumors in all three time groups.

Figure 6 shows a comparison of tumor volume curves of the PI9 and control groups. The PI9 (72 h) group showed inhibited tumor volume growth compared with the control group. However, tumor volume was almost the same as the other groups from day 42. The PI9 (24 h) and PI9 (48 h) groups showed almost the same transition as the control group.

## 3. Discussion

This study showed differences in tumor-suppression effects between different support agents containing hydrogen peroxide. KORTUC II exerts a radiosensitizing effect by increasing the level of intratumoral oxygen and decreasing peroxidase activities. When comparing our previous study and the present investigation, the methods of intratumoral injection and irradiation were identical for each group of mice, but the time periods to irradiation differed [9]. The purpose of this was to clarify whether the period of holding hydrogen peroxide differed depending on the support agent. If the period for which hydrogen peroxide can be retained is long, tumor cells under hypoxic conditions can be oxygenated for a long time, allowing therapeutic effect to be obtained with once-weekly injection. This study therefore considered whether hydrogel is a support agent that can hold hydrogen peroxide better than sodium hyaluronate.

When changes in tumor volume were compared between KORTUC groups, KORTUC (24 h) showed the greatest suppression. However, tumor-suppressive effects did not occur with longer periods before irradiation. In other words, KORTUC, which uses hydrogen peroxide and sodium hyaluronate, showed a therapeutic effect when irradiation was performed at until 48 h after injection, but none when irradiation was performed 72 h after injection. These results show that retention of oxygen in KORTUC treatment is within 48 h, and twice-weekly injection is needed.

When changes in tumor volume were compared between PI5 groups, the group showing the most tumor suppression was the PI5 (48 h) group. Moreover, the PI5 (24 h) group showed lower suppression than the PI5 (72 h) group. Although survival rates of all PI5 groups were low, only the PI5 groups showed disappearance of mouse tumors in all three time groups. Consequently, in spite of the slowest tumor growth rate of the PI5 group, the survival rate of the PI5 group was also lower. This may imply that PI5 hydrogel is cytotoxic to animals in the dose used in the study. Mice that survived to day 60 had either a small tumor or none at all. Since mice with large tumors had died by day 46, the graph of tumor volume shows a low value. When changes in the tumor volume of mice in which the tumor had not disappeared were compared between all groups, only the PI5 group showed tumor suppression. From these results, when performing New KORTUC using PI5, we think that the radiosensitization effect by hydrogen peroxide is best when irradiation is performed after 48 h. Since the period for the release of physiologically active substances by PI5 is about three weeks, irradiation after 48 h is thought to allow the full suppression effect. We thus consider PI5 as a support agent that may be able to reduce the frequency of intratumoral injection.

When changes in tumor volume were compared between PI9 groups, the greatest tumor- suppressive effect was shown in the PI9 (72 h) group. Suppression effects also tended to improve as time increased before irradiation. However, on day 46, tumor volumes of the PI9 (72 h) group reverted to almost the same as those in the other groups. Thereafter, similar changes were seen. Moreover, in the PI9 (72 h) group, two mice that survived to day 60; one with no tumor, and the other with a large tumor. The PI9 (24 h) and PI9 (48 h) groups showed almost the same transition, comparable to the control group. From these results, since PI9 takes a long time to release hydrogen peroxide, irradiation within 72 h seems likely to result in less-than-full acquisition of the radiosensitization effect. When measuring the burst size of hydrogen peroxide to PBS, the release of hydrogen peroxide was more than that of PI5. So, for irradiation within 72 h, the sensitization effect by hydrogen peroxide may hold promise. Since the PI9 (72 h) group showed tumor suppression, we think that PI9 offers a support agent that can hold hydrogen peroxide for a long time. Moreover, since the release of physiologically active substances from PI9 is about two weeks, we think that PI9 can affect radiation sensitization with once-weekly hydrogen peroxide injection.

As stated above, we think that hydrogel offers a suitable agent to replace sodium hyaluronate. However, since PI9 is a sheet-like hydrogel, a surgical procedure is required for administration to tumors. Administration to mice in these experiments required making an incision in the tumor with an 18-G needle, and placement of PI9 containing the hydrogen peroxide. Such a procedure is likely to prove unsuitable for clinical use. We thus examined how to place PI9 into tumors without surgery. Gelatin sponge as used for vascular embolization shows a composition similar to hydrogel. These gelatin sponges can be used to arrest hemorrhage and absorb sterile material. Since being developed as hemostatic materials for use in surgery, gelatin sponges have been used to affect hemostasis and decubitus ulceration. When used for vascular embolization, this sheet-like gelatin sponge can be made into a small piece with a diameter of several millimeters using a knife or scissors, and when mixed with contrast media, can be made into a gel. The material can then be injected into a given blood vessel through a vasucular catheter to interrupt blood flow. If PI9 could be used in a similar manner, placement of PI9 into tumors without surgery would be easily achieved.

In this study, the therapeutic effect was best in the KORTUC (24 h) group. However, in KORTUC, we found that the tumor-suppressive effect disappeared with prolongation of the time to irradiation. Since PI5 and PI9 showed tumor-suppressive effects with longer periods before irradiation, this implies that hydrogel is suitable as an agent to hold hydrogen peroxide for a longer time than sodium hyaluronate. Based on previous studies and the current data, we intend to continue the development of New KORTUC.

## 4. Materials and Methods

### 4.1. Animal Experiments

We performed experiments in C3H/He mice (7 weeks old, female) at the Institute for Laboratory Animal Research of Kochi Medical School. Humidity and air temperature were held constant, and animals had ad libitum access to food and water. The care and treatment of experimental animals were in accordance with institutional guidelines.

For the experiment, mice were allocated to one of ten groups (*n* = 6 in each group) according to the solutions to be injected and time periods until irradiation: control group, receiving 20 μL of phosphate-buffered saline (PBS) alone; KORTUC group, receiving 20 μL of 0.8% (*w*/*v*) sodium hyaluronate containing 0.5% (*w*/*v*) hydrogen peroxide; PI5 group, receiving PI5 hydrogel containing 20 μL of 0.5% (*w*/*v*) hydrogen peroxide; or PI9 group, receiving PI9 hydrogel containing 20 μL of 0.5% (*w*/*v*) hydrogen peroxide. For PI5, we made 2 mg of hydrogel particles containing 20 μL of 0.5% (*w*/*v*) hydrogen peroxide, and that mixture was injected into tumors. For PI9, we made 2 mg of hydrogel containing 20 μL of 0.5% (*w*/*v*) hydrogen peroxide, and that mixture was implanted surgically into tumors.

SCC VII tumor cells (approximately 10^5^) were inoculated into the right thigh of each mouse. When all tumors had grown to approximately 10 mm along the major axis, intratumoral injection of each agent was performed. At 24, 48, or 72 h following the injections, each tumor was irradiated with a 6 MeV electron beam (dose rate 1000 MU/min) using a linear accelerator (Clinac-iX, Varian Medical, Tokyo, Japan) to a total dose of 30 Gy. Irradiation was performed using our dedicated apparatus for shielding the body of the animal, excluding the tumor-bearing right thigh [17].

During the 60-day observation period, we measured changes in tumor volume using calipers and calculated the survival rate in each group. The tumor volume was calculated using the following approximate expression.

V = (W^2^ × L)/2

where V is tumor volume, W is length of the minor axis, and L is length of the major axis.

### 4.2. Measurement of Released Hydrogen Peroxide to PBS

We also measured the concentration of hydrogen peroxide released into the PBS. First, we placed 2 mg of MedGel (Hydrogel) into a sampling tube and delivered 20 μL of hydrogen peroxide dropwise. Twenty microliters of 0.5% *w*/*v* hydrogen peroxide was added to PI5 and PI9, respectively. These sampling tubes were left at room temperature for 30 min or at 4 °C overnight to completely impregnate the MedGel with the hydrogen peroxide. One milliliter of PBS was added and allowed to permeate undisturbed at 37 °C. We then measured hydrogen peroxide concentrations at 30 min and at 2, 4, 8, 24, and 48 h after adding PBS.

### 4.3. Test Agents

ARTZ^®^ dispo (Seikagaku Kogyo, Co. Ltd., Tokyo, Japan) was used as a source of sodium hyaluronate. ARTZ^®^ dispo is an injectable 1% sodium hyaluronate preparation often used for the treatment of osteoarthritis and rheumatoid arthritis affecting the knee and for the treatment of frozen shoulder [18,19]). ARTZ^®^ dispo acts through several different mechanisms: (1) protecting joint tissue by covering it and also helping to lubricate the joint; (2) soaking into cartilage and inhibiting cartilage degeneration; (3) permeating into the synovial tissue, where it inhibits inflammation and degenerative changes; and (4) inhibiting the production of pain neurotransmitters, thereby reducing pain. Through these actions, this formulation can help to improve joint function and daily life.

The MedGel (MedGEL, Co. Ltd., Tokyo, Japan) used in this experiment is a biodegradable hydrogel for the sustained release of drugs, and is a DDS (drug delivery systems) substrate that can gradually release physiologically active substances [20,21,22]. This substrate is not dissolved in water and can hold physiologically active substances by molecular interactions, primarily electrostatic, with gelatin. This hydrogel stabilizes physiologically active substances that are easily metabolized and inactivated in vivo, but is decomposed by degradative enzymes (e.g., collagenase), resulting in release of the biologically active substance in a controlled, sustained fashion. Three kinds of MedGel are available (PI5, PI9, and E50), and the kind of gelatin used as a raw material differs in each. Given the gelatin used, the optimal hydrogel should be selected based on the electric charge of the physiologically active substance to be delivered. PI5 is suitable for positive charges, and PI9 is suitable for negative charges. E50 is used to chemically introduce a cation group into the gelatin in order to release substances with strong negative charges, such as nucleic acids (DNA/RNA). Since the slow release of hydrogen peroxide was our goal, PI5 and PI9 were used in the present study.

## 5. Conclusions

The goal of this study was thus to develop a new formulation of KORTUC (New KORTUC) that only requires once-weekly administration as a radiosensitizer. New KORTUC, which contains hydrogen peroxide and hydrogel, exerted a radiosensitizing effect that persisted beyond 72 h following injection of the agent. Use of this new formulation allows radiosensitizer injections to be performed once-weekly with good effect.
